# Colour lightness of butterfly assemblages across North America and Europe

**DOI:** 10.1038/s41598-018-36761-x

**Published:** 2019-02-11

**Authors:** Pablo Stelbrink, Stefan Pinkert, Stefan Brunzel, Jeremy Kerr, Christopher W. Wheat, Roland Brandl, Dirk Zeuss

**Affiliations:** 1 Faculty of Biology, Department of Ecology – Animal Ecology, Philipps-Universität Marburg, Karl-von-Frisch-Strasse 8, 35043 Marburg, Germany; 2 Faculty of Landscape Architecture, Horticulture and Forestry, Department of Biodiversity and Species Conservation, University of Applied Science Erfurt, Leipziger Strasse 77, 99085 Erfurt, Germany; 3 Department of Biology, University of Ottawa, Ottawa, Canada; 4 Department of Zoology, Stockholm University, 10691 Stockholm, Sweden

## Abstract

Melanin-based dark colouration is beneficial for insects as it increases the absorption of solar energy and protects against pathogens. Thus, it is expected that insect colouration is darker in colder regions and in regions with high humidity, where it is assumed that pathogen pressure is highest. These relationships between colour lightness, insect distribution, and climate between taxa and subtaxa across continents have never been tested and compared. Here we analysed the colour lightness of nearly all butterfly species of North America and Europe using the average colour lightness of species occurring within 50 km × 50 km grid cells across both continents as the dependent variable and average insolation, temperature and humidity within grid cells as explanatory variables. We compared the direction, strength and shape of these relationships between butterfly families and continents. On both continents, butterfly assemblages in colder and more humid regions were generally darker coloured than assemblages in warmer and less humid regions. Although these relationships differed in detail between families, overall trends within families on both continents were similar. Our results add further support for the importance of insect colour lightness as a mechanistic adaptation to climate that influences biogeographical patterns of species distributions.

## Introduction

Colouration is a fundamental feature of organisms, and understanding its variation among species has fascinated scientists since the beginning of natural history^1–3^. Several biotic and abiotic processes have been proposed to explain the different aspects of animal colouration, i.e. colour (chroma), colour patterns and colour lightness. It is expected that variation in the colours and colour patterns of species are primarily driven by biotic factors via signalling related to cryptic colouration^4,5^, aposematism^6^ and sexual selection^7,8^. By contrast, it is likely that abiotic factors primarily drive variation in the colour lightness of species, which describes the amount of reflected solar radiation irrespective of its wavelength. In turn, colour lightness probably correlates with temperature gradients because of its role in thermoregulation^9,10^. However, because colour lightness is primarily regulated by melanin pigmentation levels, and melanin protects against UV radiation^11,12^ and pathogens^13^, colour lightness might also correlate with additional environmental gradients.

The vast majority of species on Earth are insects, which rely upon external energy to achieve optimal body temperatures necessary for physiological processes and behaviour^14,15^. For insects to achieve functional body temperatures, it is expected that they have lower colour lightness (i.e. are darker coloured) in colder climates because higher concentrations of melanin in the cuticle increase the absorption of solar energy^9,16,17^. This should be especially important for heliothermic insects. On the other hand, insects should have a higher colour lightness (i.e. are lighter coloured) in warmer climates to reduce the risk of overheating^16,18^. A wide range of studies empirically support this hypothesis of thermal melanism of insects^18–28^.

In addition to thermoregulatory demands, other functions of melanin-based colouration might also affect geographical patterns of insect colour lightness. First, high concentrations of melanin in the cuticle protect the genome from detrimental effects of UV radiation^12^. Second, melanin plays an important role in the immune system of insects, indirectly through increasing the structural integrity of cells, which improves resistance to pathogens, as well as directly through the phenoloxidase-activated response to infection and parasitism^13,29–32^. Thus, if melanism provides protection against UV radiation, we should expect to observe a large-scale decrease of insect colour lightness with increasing solar radiation, which is in contrast to the prediction of the thermal melanism hypothesis. Additionally, if melanism provides protection against pathogens, we should expect to observe decreased colour lightness of insects in humid environments, where pathogen pressure is likely highest^33–35^.

Here, we considered nearly all the butterfly species of North America and Europe to test the above-mentioned links between colour lightness, distribution of species and climate. A number of studies on single butterfly species in different regions of the world and a macroecological study on European butterfly species already support the hypothesis that thermal melanism is generally important for this group of insects^10,16,24,28,36–43^. However, whether these results also hold for the butterfly fauna of North America and whether trends are consistent across butterfly families and continents remains unexplored.

North America and Europe experienced different biogeographical histories since their separation about 200 Ma ago^44–46^. Today, they differ in their topography, with mountain ranges mainly oriented in the north–south direction in North America and mainly oriented in the east–west direction in Europe. The geographical setting of Europe has limited the colonization of northern areas by tropical lineages, whereas North America has had a longer continuous connection to the tropics and its temperate and boreal zones, which has facilitated the dispersal of species from tropical and subtropical regions. Similar geographical patterns of insect colour lightness on both continents and across families would add further support for colour lightness as a mechanistic adaptation of insects to climate, which influences biogeographical patterns of species distributions.

We tested the predictions that butterfly assemblages in both North America and Europe (1) have a lower colour lightness in cooler climates and a higher colour lightness in warmer climates as expected by the thermal melanism hypothesis, and (2) have a lower colour lightness in regions with high solar radiation and a higher colour lightness in regions with low solar radiation because of the protective function of melanin against damage from UV radiation. Additionally, we tested the prediction that (3) butterfly assemblages across North America and Europe have a lower colour lightness in more humid environments to protect against higher putative pathogen pressures in these areas. Finally, we tested whether these predictions also hold on both continents at the level of butterfly families.

## Methods

To test the hypothesized links between insect colour lightness, distribution of species, climate across North America and Europe, and butterfly families, we combined data on the distribution and colour lightness of 330 North American and 326 European butterfly species. For our analysis, we used an assemblage-based approach that allows the combination of distributional and trait information with environmental information by aggregating traits of co-occurring species and environmental variables in a spatially explicit context. We created a 50 km × 50 km grid of North America (North America Albers equal area projection; EPSG: 102008) with 9,220 cells covering ca. 23 million km². We also used a 50 km × 50 km grid of Europe (Europe Albers equal area projection; EPSG: 102013) with 1,939 cells covering ca. 5 million km². For each grid cell, we calculated the average colour lightness of all co-occurring species and average climatic conditions from global datasets (see below).

### Species

We analysed four species-rich butterfly families (Lycaenidae, Nymphalidae, Papilionidae, Pieridae), which include most North American and European species. For comparability, we excluded Hesperiidae because they are depicted in a slightly different posture in the data source for North America. Family affinity followed published classifications for North American^47^ and European^48^ taxa. We grouped 34 North American butterfly species into 13 species complexes to account for inconsistent taxonomic classifications of species or subspecies (Appendix S1 in Supporting Information). Trait data of these North American species were averaged for each species complex. For lists of species and family classifications, see Appendices S2 and S3.

### Colour lightness data

The colour lightness of each butterfly species was calculated using computer-assisted digital image analysis following protocols previously described in ref.^10^ (also applied in, e.g. refs^26,28,49^). We scanned the dorsal and ventral images of North American butterfly species in ref.^47^ with a resolution of 1,200 dpi and 24 bit in the RGB colour space. Analyses focused upon the body and 1/3 of the wing area closest to the body because this area is probably the most important for thermoregulation^50^. Images were cropped with Adobe Photoshop CS2. Only images of females and monomorphic species were used to exclude potential effects of sexual dichromatism^2,8,51–53^. We computed colour lightness values as the average of the red, green and blue colour channels of the considered area using the R package *EBImage*^54^. The resulting values range from 0 (black) to 255 (white). The colour lightness of European butterfly species was taken from ref.^10^. We included in the analyses only those species for which images of both the ventral and dorsal sides were available. We used one image per species as presented in the data sources, thereby ignoring intraspecific variability. If different morphs were depicted, we averaged their colour lightness to obtain one value per species. However, note that colour lightness values of species that occur in both North America and Europe but whose images were obtained from independent sources (refs^47,48^) were highly correlated with a slope of 1 (p < 0.001, *r*² > 0.96, n = 16 species, Appendix S4), thus providing an important cross validation of the two datasets used in our approach.

### Distribution data

Distribution data for butterflies of North America were obtained from published contour maps^55^, which are based on expert knowledge and extensive data collections. These contour maps were digitized and then processed into presence data for the grid of North America using the R package *rgdal*^56^. Distribution data for butterflies of Europe were obtained from ref.^10^, originally available as atlas data of 50 km × 50 km resolution^57^. For North America, 435 species (Europe: 393 species) of the families Lycaenidae, Nymphalidae, Papilionidae and Pieridae with images were available, out of which 330 species (Europe: 326 species) remained after matching the colour lightness data (species depicted both with the ventral and the dorsal side) with the distribution data. We included only grid cells with at least five species in our analyses to stabilize the estimates of the average colour lightness within grid cells and to be consistent with previous work (e.g. refs^10,26,49^). Thereby, the number of grid cells used in our analyses was reduced to 8,499 in North America and 1,840 in Europe without substantially decreasing the number of species in the dataset (Appendix S5).

### Environmental variables

Environmental variables were assembled from *microclim*, which provides global hourly data for an average day of each month of the year^58^. We calculated annual averages of the variables solar insolation (INS, Wm^−2^), air temperature at 1.2 m above ground (TMP, °C), and relative humidity at 1.2 m above ground (HUM, %). These data were obtained and aggregated for each grid cell using functions of the R package *raster*^59^. These explicit variables have the advantage that statistical results can be directly compared between continents, in contrast to previous work that used principal components as predictors (ref.^10^). Maps of the environmental variables can be found in Appendix S6.

### Statistical analysis

We used ordinary least-squares regressions (OLS) to test for relationships between the average colour lightness of butterfly assemblages and the environmental variables. Models were calculated for each continent and differentiated according to family and dorsal or ventral side. We calculated these models also with quadratic predictors to account for potential non-linear relationships. We assumed a quadratic relationship if the difference between the *r*² values of the quadratic and linear model was larger than 0.1 (Δ*r*² = quadratic *r*² − linear *r*²).

Spatial autocorrelation can lead to an overestimation of the degrees of freedom owing to the inherent non-independence of the neighbouring grid cells and hence to false parameter estimates and model inference^60^. In addition to the OLS models, we therefore also calculated generalized least-squares model (GLS) in which we account for autocorrelation in the model errors using a Gaussian spatial correlation structure of geographical coordinates (Manhattan distance metric).

We used Tukey HSD tests (R package *agricolae*^61^) to assess differences in dorsal and ventral colour lightness between families and continents.

## Results

### Geographical patterns

**Figure 1:**
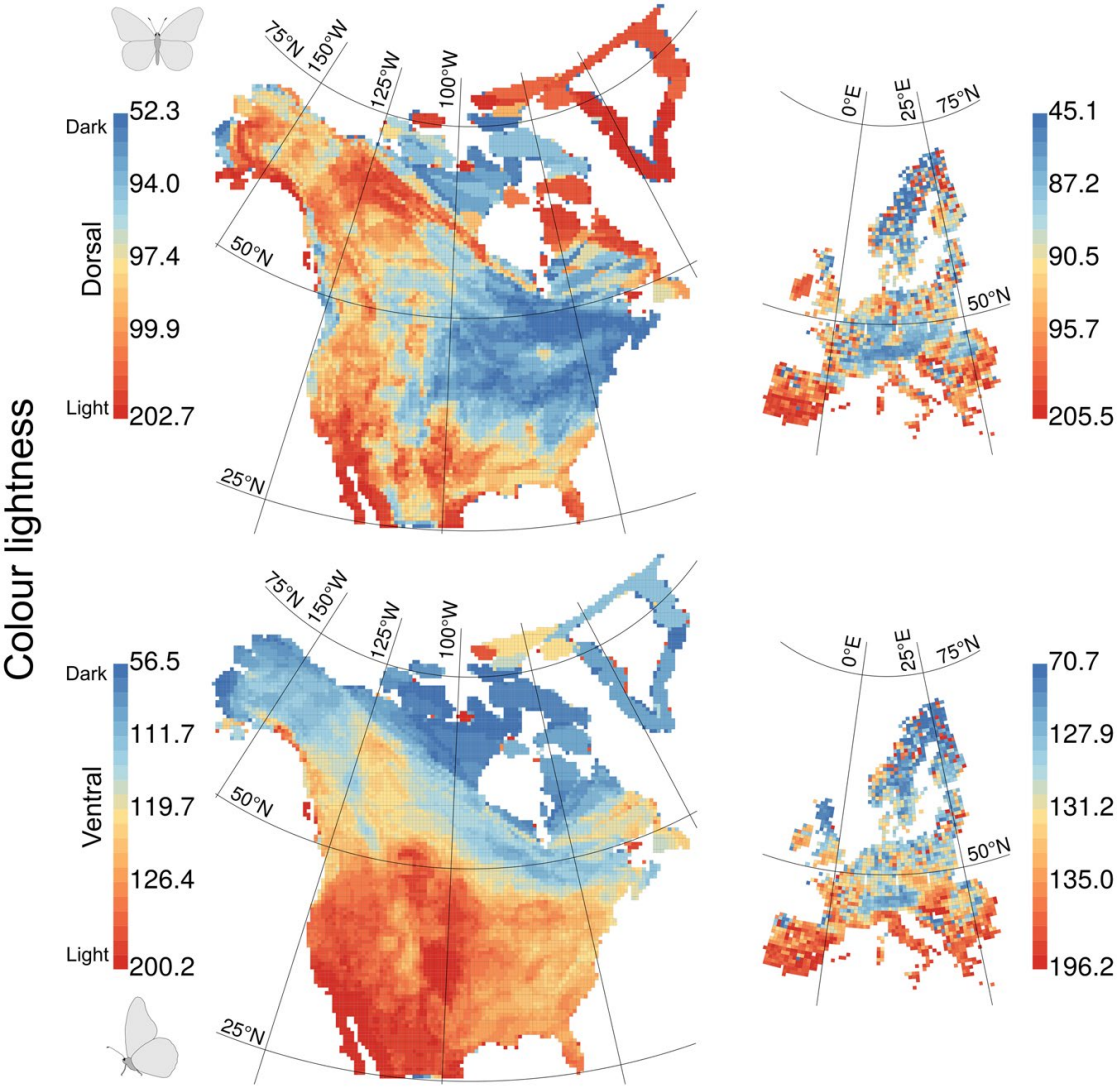
Average colour lightness of butterfly assemblages of all species in North America and Europe. Colour lightness ranges from 0 (black) to 255 (white) and was categorized using quantiles, with red indicating light-coloured assemblages and blue indicating dark-coloured assemblages. North American data are mapped on a North America Albers equal area conic projection (EPSG: 102008; 330 species across 9,220 grid cells); European data are mapped on a Europe Albers equal area conic projection (EPSG: 102013; 326 species across 1,939 grid cells).

**Figure 2:**
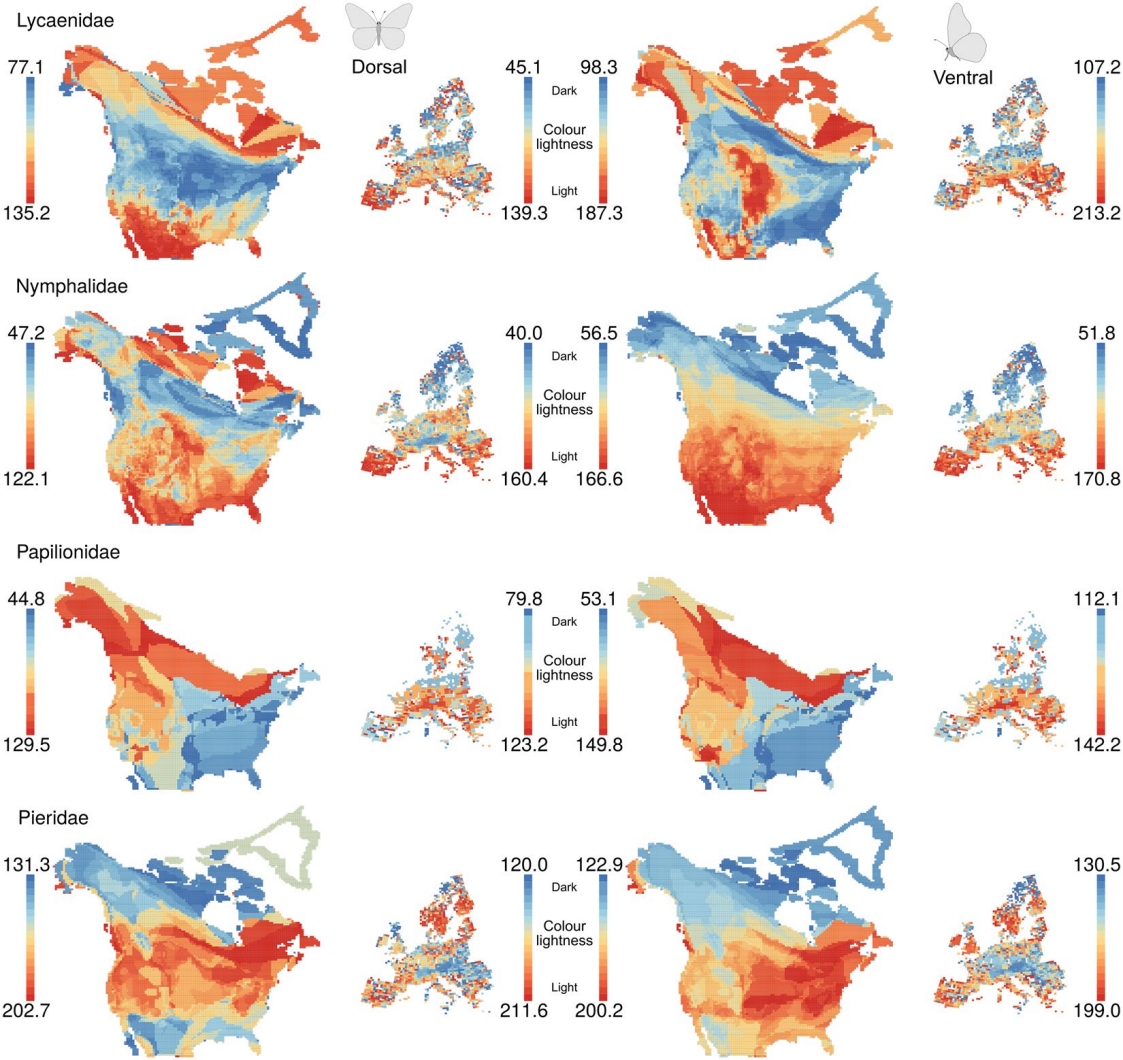
Average colour lightness of families of butterfly assemblages in North America and Europe. Colour lightness ranges from 0 (black) to 255 (white) and was categorized using quantiles, with red indicating light-coloured assemblages and blue indicating dark-coloured assemblages.

North American butterfly assemblages in the eastern mainland were on average darker coloured on the dorsal side, and those in northern regions were on average darker coloured on the ventral side. European butterfly assemblages in northern regions were on average darker coloured on both the dorsal and ventral sides (Fig. 1). The geographical distribution of colour lightness on both continents differed between families and between the dorsal and ventral sides of the families (Fig. 2). Especially the ventral side of Nymphalid assemblages was clearly darker coloured in the north and lighter coloured in the south on both continents (Fig. 2).

### Statistical analyses

**Figure 3:**
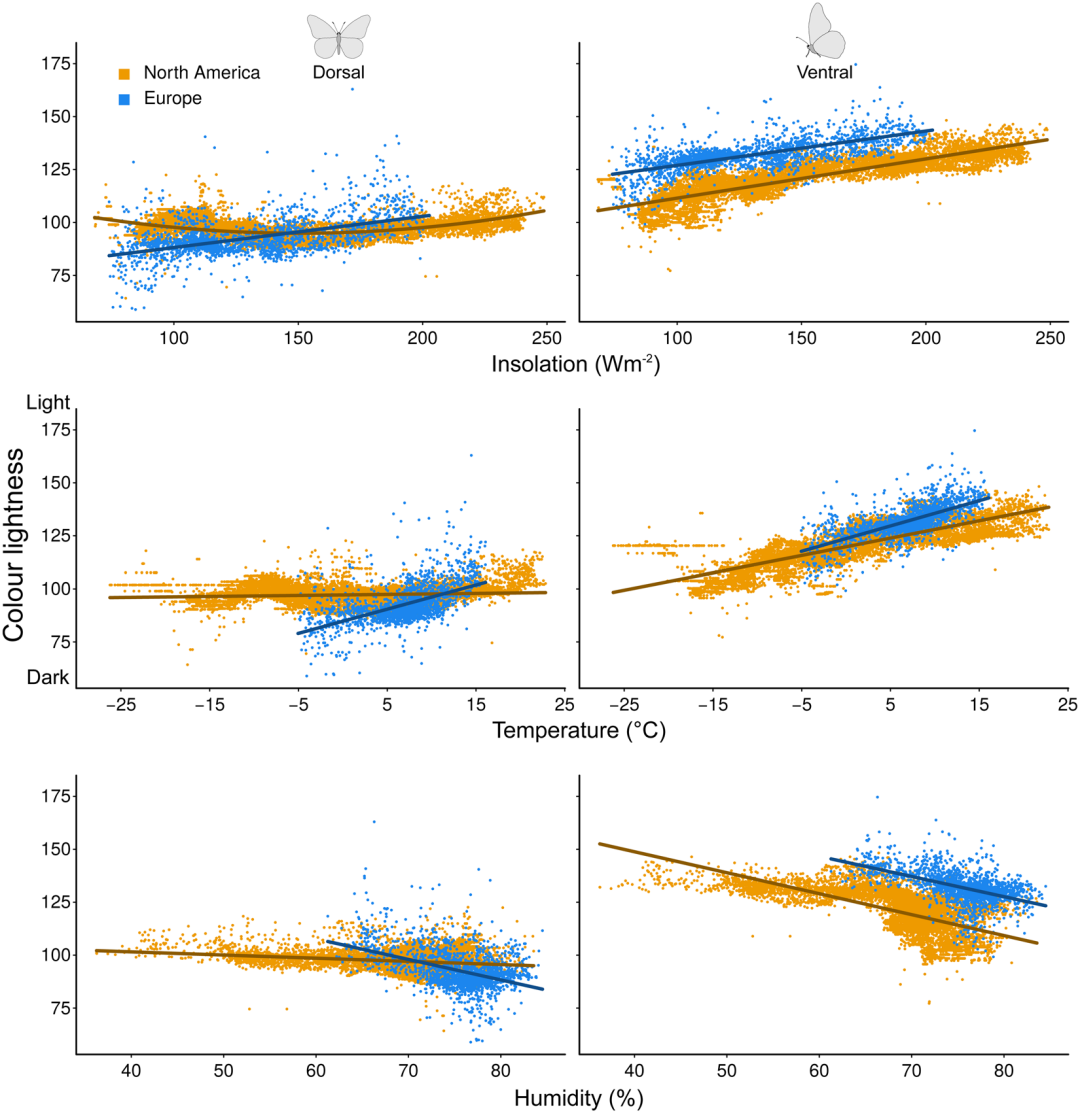
Scatterplots showing the correlation between average colour lightness of butterfly assemblages in North America and Europe and environmental variables. Colour lightness ranges from 0 (black) to 255 (white). Insolation and temperature were included to test for the putative thermoregulatory and UV protection function of insect colour lightness, and humidity was included to test for the putative pathogen protection function of insect colour lightness. Note that colour lightness increased with insolation and temperature and decreased with humidity in both North America and Europe.

Overall, dorsal and ventral butterfly colour lightness in assemblages was positively correlated with insolation and temperature and negatively correlated with humidity in both North America and Europe. While these relationships were linear in Europe, we found a U-shaped relationship between dorsal colour lightness and insolation and a weak linear dependence on temperature in North America (Fig. 3, Table 1). However, ventral colour lightness in North America was strongly positively correlated with insolation and temperature (linear shape; INS: *r*² = 0.71, p < 0.001, TMP: *r*² = 0.64, p < 0.001). All three environmental variables consistently explained more variance in ventral colour lightness than in dorsal colour lightness on both continents (Table 1).

**Table 1:** Statistics of linear and quadratic ordinary least-squares regressions between the colour lightness of butterfly assemblages across North America and Europe and the three potential environmental drivers temperature, insolation and humidity.

Variable	Family	Side	North America				Europe			
			Linear *r*²	Quadratic *r*²	Δ *r*²	Shape	Linear *r*²	Quadratic *r*²	Δ *r*²	Shape
INSOLATION	All	Dorsal	+0.01	**+0.18**	0.17		+0.25	+0.27	0.02	
		Ventral	**+0.71**	−0.72	0.01		+0.45	n.s.	0.00	
	Lycaenidae	Dorsal	+0.03	+ 0.32	0.29		**+0.13**	+0.14	0.01	
		Ventral	−0.00	+0.05	0.05		**+0.09**	−0.13	0.04	
	Nymphalidae	Dorsal	+0.11	+0.19	0.08		+0.42	+0.43	0.01	
		Ventral	**+0.90**	−0.92	0.02		**+0.55**	−0.56	0.01	
	Papilionidae	Dorsal	−0.01	+0.07	0.06		−0.15	n.s.	0.00	
		Ventral	n.s.	+0.01	0.01		−0.12	n.s.	0.00	
	Pieridae	Dorsal	+0.01	**−0.47**	0.46		n.s.	**+0.10**	0.10	
		Ventral	+0.30	**−0.70**	0.40		n.s.	**+0.13**	0.12	
TEMPERATURE	All	Dorsal	+0.01	+0.09	0.08		**+0.28**	+0.34	0.06	
		Ventral	+0.64	−0.64	0.00		**+0.46**	+0.47	0.01	
	Lycaenidae	Dorsal	+0.05	**+0.38**	0.33		+0.12	+0.17	0.05	
		Ventral	**−0.03**	+0.03	0.00		+0.04	n.s.	0.00	
	Nymphalidae	Dorsal	+0.09	+**0.23**	0.14		**+0.45**	+0.51	0.06	
		Ventral	+0.84	−0.85	0.01		+0.54	+0.55	0.01	
	Papilionidae	Dorsal	**−0.58**	+0.61	0.03		**−0.29**	n.s.	0.00	
		Ventral	**−0.51**	+0.55	0.04		**−0.33**	n.s.	0.00	
	Pieridae	Dorsal	+0.00	−0.44	0.44		n.s.	+0.03	0.03	
		Ventral	+0.39	−0.64	0.25		+0.02	+0.05	0.03	
HUMIDITY	All	Dorsal	−0.04	+0.06	0.02		−0.19	+0.25	0.06	
		Ventral	−0.41	−0.44	0.03		−0.27	+0.28	0.01	
	Lycaenidae	Dorsal	− 0.06	+0.13	0.07		−0.11	+0.11	0.00	
		Ventral	−0.01	−0.02	0.01		−0.07	−0.09	0.02	
	Nymphalidae	Dorsal	−0.05	+0.06	0.01		− 0.29	+0.32	0.03	
		Ventral	−0.45	−0.47	0.02		−0.33	n.s.	0.00	
	Papilionidae	Dorsal	−0.29	−0.40	0.11		+0.15	n.s.	0.03	
		Ventral	−0.32	−0.38	0.06		+0.13	n.s.	0.01	
	Pieridae	Dorsal	+0.01	−0.03	0.02		n.s.	+0.02	0.02	
		Ventral	−0.00	−0.01	0.01		n.s.	+0.07	0.07	

Models were computed for the complete datasets of each continent and separately for the major butterfly families. Direction of the effects (+/−) and *r*² values are given for each model. If Δ*r*² (quadratic *r*² − linear *r*²) was >0.1, a quadratic shape (U- or hump-shaped) of the relationship was assumed; otherwise, a linear shape was assumed as also indicated by the inserted pictograms. Only assemblages with at least five species were included in this analysis. Level of significance was set to 0.001 (n.s. = not significant). Highest *r*² values fo*r* the assumed relationship in each family and continent are highlighted in bold.

In Europe, the colour lightness of assemblages of Lycaenids and Nymphalids increased with insolation and temperature and decreased with humidity on both the dorsal and ventral side (Table 1, Appendix S7). In North America, ventral colour lightness of Nymphalids was strongly positively correlated with insolation and temperature (linear shape; INS: *r*² = 0.90, p < 0.001, TMP: *r*² = 0.84, p < 0.001), whereas ventral colour lightness of Lycaenids was weakly negatively correlated with insolation and temperature. We found a U-shaped relationship with insolation and temperature for dorsal colour lightness of both North American Lycaenids and Nymphalids. By contrast, colour lightness of Papilionids was strongly negatively correlated with temperature in both North America and Europe and negatively correlated with insolation and positively correlated with humidity in Europe. We found strong hump-shaped relationships between the colour lightness of Pierids in North America and insolation as well as temperature (hump shape; INS: dorsal: *r*² = 0.47, p < 0.001, ventral: *r*² = 0.70, p < 0.001). The colour lightness of Pierid assemblages in Europe was not significantly or only weakly positively correlated with temperature, but the colour lightness of the ventral side showed a hump-shaped relationship to insolation. Humidity was a weak predictor of the colour lightness of Pierid assemblages on both continents (Table 1, Appendix S7). Results of the GLS analysis to account for spatial autocorrelation were very similar to the above-mentioned results (Appendix S8). We therefore conclude that spatial autocorrelation does not affect our main findings.

**Figure 4:**
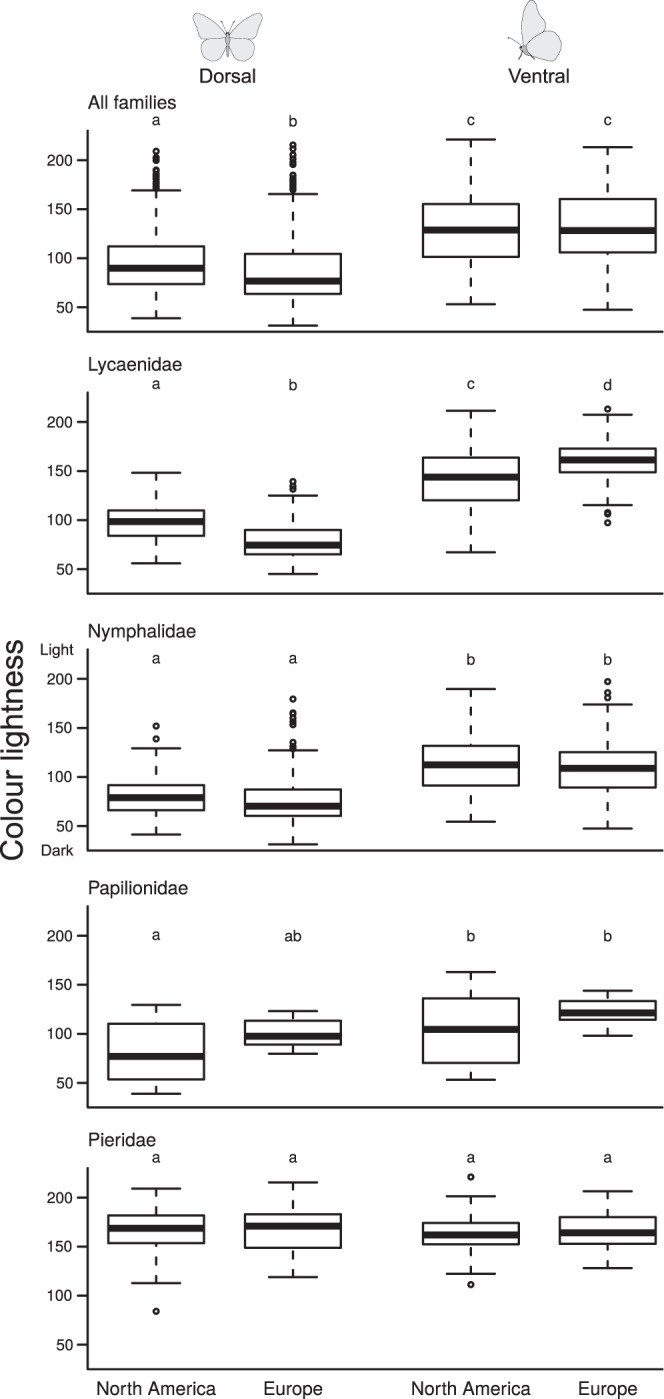
Colour lightness of dorsal and ventral sides of North American and European butterfly species. Boxplots are given for the dorsal and ventral colour lightness of all species combined and separately for four butterfly families. Colour lightness ranges from 0 (black) to 255 (white). Note that the dorsal side tends to be darker coloured than the ventral side in all families except for Pieridae.

Overall, butterfly species were on average darker coloured on the dorsal side than on the ventral side on both continents (Fig. 4; North America: dorsal 98.12, ventral 127.55, p < 0.001; Europe: dorsal 89.22, ventral 130.95, p < 0.001). Within families, this result was also found for Lycaenidae and Nymphalidae, and for Papilionidae only in North America; for Pieridae, there was no significant difference between dorsal and ventral colour lightness (Fig. 4, Appendix S9). For the distribution of colour lightness values within and across families, see Appendix S10.

## Discussion

Our results showed that insolation and temperature are important environmental drivers of geographical patterns of the colour lightness of butterfly assemblages across both North America and Europe. Butterfly assemblages in colder regions of both continents were consistently on average darker coloured than assemblages in warmer regions. Thus, our results add further macroecological support for the thermal melanism hypothesis for butterflies of the Northern Hemisphere and demonstrate that biogeographical patterns of colour lightness of the butterfly fauna of North America are broadly similar to those of Europe. Furthermore, the average colour lightness of butterfly assemblages decreased with increasing humidity on both continents, although the relative importance of humidity as an explanatory variable was generally lower than that of insolation and temperature. Nevertheless, this finding points to additional benefits of being dark in order to protect against pathogens. However, being dark was not relevant for the butterfly faunas of North America and Europe for protecting against UV radiation, as assemblages were on average lighter coloured in areas with high insolation, not darker coloured. It is important to note that our study presents a macroecological perspective and that the relative importance of functions associated with colour lightness might be different when viewed at different spatial scales.

Even though our findings of the colour lightness of North American and European butterfly assemblages were basically similar at the level of the butterfly families considered, there were also notable differences between continents and families.

First, the relationships between environmental variables and the average colour lightness of butterfly assemblages tended to be more curvilinear in North America than in Europe. This difference might partly be caused by the larger environmental gradients in North America than in Europe, which might lead to a change in the relative importance of functions associated with colour lightness at more extreme environmental conditions. For Pierids in North America, for example, average colour lightness increased with insolation and temperature, but then again tended to decrease under very high levels of solar radiation (see also Appendix S7). This change might be due to a shift in the relative importance of thermoregulation for protection from UV radiation under very high levels of solar radiation, as for example suggested by ref.^25^. Furthermore, a considerable proportion of North America lies north of the polar circle, where species receive very high amounts of insolation during summer, although the annual average insolation as captured by our variable is comparably low. This geographical characteristic might have blurred the relationship between average colour lightness of butterfly assemblages and climatic variables and might furthermore be responsible for the relatively large share of light-coloured species in northern parts of North America. Ideally, future research could use species-specific and spatially-explicit environmental variables during the activity period of adults to further elucidate the climate-colour lightness relationship once the data is available.

Second, the colour lightness of Papilionids decreased with increasing insolation and temperature in contrast to the other families, i.e. Papilionids were on average lighter coloured in northern areas than in southern areas of both North America and Europe. Notably, Papilionid species are on average larger than species of the other butterfly families examined here^62,63^. In colder environments, it thus might be more efficient for Papilionids to absorb long-wave thermal energy by pressing their bodies to warm surfaces and to invest resources in their body size instead of melanin synthesis.

Third, Papilionids and Pierids, whose geographical patterns of colour lightness deviated most from the expectations based on thermal melanism and protection from pathogens, had much lower diversification rates in their evolutionary history compared to Lycaenids and Nymphalids^64^. It thus seems possible that thermoregulatory adaptations related to the colour lightness of Lycaenids and Nymphalids provided an evolutionary advantage for the colonization of temperate and boreal climates, which could have ultimately led to their diversification in the Northern Hemisphere. This idea is supported by higher species numbers of Lycaenids and Nymphalids in both North America and Europe compared to those of Papilionids and Pierids. Such conservatism of the evolution of thermal adaptations has already been shown to shape the pattern of phylogenetic diversity of European dragonfly assemblages^65^. However, the role of colour lightness for the diversification of butterflies and insects in general remains speculative until comprehensive phylogenies and colouration data are available.

An interesting result emerging from our study is that butterflies in the families Lycaenidae, Nymphalidae and Papilionidae were on average darker coloured on the dorsal side than on the ventral side (Fig. 4). This phenomenon is known as countershading and plays an important role in crypsis^66,67^. Directional illumination from the sun causes shadows on the ventral side of organisms, and the resulting gradients in colour lightness are used as visual cues by predators to detect prey. Relatively light-coloured ventral sides reduce this shadowing and render organisms less conspicuous. If countershading really is important in butterflies of the Northern Hemisphere as our results suggest, then this would have implications for their basking behaviour — butterflies would predominantly use their darker-coloured dorsal side for absorbing solar energy in colder environments and their lighter-coloured ventral side for reflecting solar energy in warmer environments. This would allow them to use colour lightness for countershading and thermoregulation simultaneously.

Despite the clear patterns that emerged from our data sets, several important questions for future colour lightness research remain (1) Can the consistent relationship between insect colour lightness and the thermal environment be used to improve species distribution modelling? Given that insolation explained 90% of the variance in ventral colour lightness of Nymphalids (Table 1), this seems highly promising, at least for this group of butterflies. (2) Can colour lightness calculated from images be used to infer radiative heat transfer in insects? Recent results on the thermal biology of unicellular fungi not only indicate that energy harvesting through melanin-based colouration is deeply rooted in eukaryotic life, but also that average RGB colour values can be reliable proxies for heating rates^68,69^. (3) Is insect colour lightness also related to seasonal changes in the thermal environment, with for example light-coloured species predominantly occurring in warmer months of the year, and would such a relationship also be mirrored by the abundance of species?

In summary, our results showed that butterfly assemblages in colder and more humid regions were generally darker coloured than assemblages in warmer and less humid regions in both North America and Europe. Although these relationships differed in detail between families, overall trends within families on the two continents were similar. Our results thus add further support for the importance of insect colour lightness as a mechanistic adaptation to climate, which influences biogeographical patterns of species distributions.

## Electronic supplementary material

Supplementary Information

## Data Availability

All relevant data are available from the corresponding author upon request.
