# Supplementary material for: Colour lightness of butterfly assemblages across North America and Europe
Source: Sci Rep. 2019 Feb 11;9:1760. doi: 10.1038/s41598-018-36761-x (PMC6370790; doi:10.1038/s41598-018-36761-x)
Supplement: Supplementary file 1 — Supplementary Information [file 41598_2018_36761_MOESM1_ESM.pdf]

Colour lightness of butterfly assemblages across North America and Europe

– Supporting Information –

Authors

Pablo Stelbrink[1], Stefan Pinkert[1,2], Stefan Brunzel[2], Jeremy Kerr[3], Christopher W. Wheat[4], Roland Brandl[1] and Dirk Zeuss[1,4,*]

Affiliations

[1]Faculty of Biology, Department of Ecology – Animal Ecology, Philipps-Universität Marburg, Karl-von-Frisch-Strasse 8, 35043 Marburg, Germany.

[2]Faculty of Landscape Architecture, Horticulture and Forestry, Department of Biodiversity and Species Conservation, University of Applied Science Erfurt, Leipziger Strasse 77, 99085 Erfurt, Germany.

[3]Department of Biology, University of Ottawa, Ottawa, Canada.

[4]Department of Zoology, Stockholm University, 10691 Stockholm, Sweden.

[*]To whom correspondence should be addressed. Dirk Zeuss (dirk.zeuss@biologie.uni-marburg.de).

Appendix S1: Editing of databases.

Editing of databases was necessary because of different taxonomic classifications in Brock *et al.* (2003, colouration data, ref. 47) and Scott (1997, distribution data, ref. 55).

a) Seven contour maps that contained distribution data of two or more subspecies that are classified as species in colouration data were split.

Split distribution maps	Assigned colouration data
Aneae troglodyta ssp. *aidea*, *floridalis*	*Aneae aidea*<br>*Aneae troglodyta*
Anthanassa frisia ssp. *frisia*, *tulcis*	*Anthanassa frisia*<br>*Anthanassa tulcis*
Colias scudderi ssp. *gigantea*, *scudderi*	*Colias gigantea*<br>*Colias scudderi*
Erora laeta	*Erora laeta*<br>*Erora quaderna*
Fixenia favonius ssp. *ontario*, *autolycus*, *ilavia*	*Satyrium favonius* (part)<br>*Satyrium ilavia*
Papilio glaucus ssp. *canadensis*, *glaucus*	*Papilio canadensis*<br>*Papilio glaucus*
Plebejus glandon	*Plebejus glandon*<br>*Plebejus podarce*

b) Contour maps of species or subspecies that were combined to assign colouration data of one species.

Combined contour maps	Assigned colouration data
Chlosyne acastus ssp. *neumogeni*, *sabina*<br>*Chlosyne acastus* ssp. *acastus*, *vallismortis*, *dorothyi*	*Chlosyne acastus*
Chlosyne gryneus ssp. *gryneus*, *sweadneri*<br>*Chlosyne gryneus* ssp. *siva*<br>*Chlosyne gryneus* three ssp. with brown US of HW<br>*Chlosyne gryneus* ssp. *thornei*, *loki*	*Chlosyne gryneus*
Euphydryas chalcedona long-valva-prong ssp.<br>*Euphydryas chalcedona* short-valva-prong ssp.	*Euphydryas chalcedona*
Fixenia favonius ssp. *favonius*<br>*Fixenia favonius* ssp. *ontario*, *autolycus* (*ilavia* excluded)	*Satyrium favonius*
Papilio machaon northern ssp.<br>*Papilio machaon* southern ssp.	*Papilio machaon*
Polygonia gracilis ssp. *gracilis*<br>*Polygonia gracilis* ssp. *zephyrus*	*Polygonia gracilis*

c) Colouration that were averaged to be assigned to the distribution data of a species complex:

Species complex	Assigned colouration data
Apodemia mormo complex	*Apodemia mormo*<br>*Apodemia mejicanus*<br>*Apodemia virgulti*<br>*Apodemia duryi*
Callophrys affinis complex	*Callophrys affinis*<br>*Callophrys perplexa*
Celastrina ladon complex	*Celastrina ladon*<br>*Celastrina neglecta*<br>*Celastrina idella*<br>*Celastrina neglectamajor*
Colias alexandra complex	*Colias alexandra*<br>*Colias christina*
Colias hecla complex	*Colias hecla*<br>*Colias canadiensis*
Colias nastes complex	*Colias nastes*<br>*Colias tyche*
Erebia disa complex	*Erebia disa*<br>*Erebia mancinus*
Erebia mackinleyensis complex	*Erebia mackinleyensis*<br>*Erebia magdalena*
Euphilotes battoides complex	*Euphilotes battoides*<br>*Euphilotes baueri*<br>*Euphilotes bernadino*<br>*Euphilotes ellisi*<br>*Euphilotes glaucon*
Euphilotes enoptes complex	*Euphilotes enoptes*<br>*Euphilotes mojave*<br>*Euphilotes ancilla*
Neonympha aerolata complex	*Neonympha aerolata*<br>*Neonympha helicta*
Pieris oleracea complex	*Pieris oleracea*<br>*Pieris marginalis*
Speyeria atlantis complex	*Speyeria atlantis*<br>*Speyeria hesperis*

Appendix S2: List of butterfly species of North America.

**Lycaenidae**	Plebejus icarioides	Caria ino	Nymphalis antiopa	Papilio troilus
Atlides halesus	Plebejus idas	Cercyonis meadii	Nymphalis californica	Papilio zelicaon
Callophrys affinis	Plebejus lupinus	Cercyonis oetus	Nymphalis vaualbum	Parnassius smintheus
Callophrys augustinus	Plebejus melissa	Cercyonis pegala	Oeneis alberta	
Callophrys eryphon	Plebejus neurona	Cercyonis sthenele	Oeneis bore	**Pieridae**
Callophrys fotis	Plebejus optilete	Chlosyne acastus	Oeneis chryxus	Abaeis nicippe
Callophrys gryneus	Plebejus podarce	Chlosyne californica	Oeneis jutta	Anteos clorinde
Callophrys henrici	Plebejus saepiolus	Chlosyne cyneas	Oeneis macounii	Anteos maerula
Callophrys hesseli	Plebejus shasta	Chlosyne definita	Oeneis melissa	Anthocharis cethura
Callophrys irus	Satyrium acadica	Chlosyne fulvia	Oeneis nevadensis	Anthocharis lanceolata
Callophrys johnsoni	Satyrium auretorum	Chlosyne gabbii	Oeneis polixenes	Anthocharis midea
Callophrys lanoraieensis	Satyrium behrii	Chlosyne gorgone	Oeneis uhleri	Anthocharis sara
Callophrys mcfarlandi	Satyrium calanus	Chlosyne harrisii	Paramacera allyni	Aphrissa statira
Callophrys mossii	Satyrium californica	Chlosyne hoffmanni	Phyciodes batesii	Ascia monuste
Callophrys niphon	Satyrium caryaevorus	Chlosyne janais	Phyciodes cocyta	Colias alexandra
Callophrys perplexa	Satyrium edwardsii	Chlosyne lacinia	Phyciodes graphica	Colias behrii
Callophrys polios	Satyrium favonius	Chlosyne leanira	Phyciodes mylitta	Colias canadiensis
Callophrys sherdanii	Satyrium fuliginosa	Chlosyne nycteis	Phyciodes orseis	Colias christina
Callophrys spinetorum	Satyrium ilavia	Chlosyne palla	Phyciodes pallida	Colias eurytheme
Callophrys xami	Satyrium kingi	Chlosyne theona	Phyciodes phaon	Colias gigantea
Calycopis cecrops	Satyrium liparops	Chlosyne whitneyi	Phyciodes picta	Colias harfordii
Calycopis isobeon	Satyrium polingi	Coenonympha haydenii	Phyciodes pulchella	Colias hecla
Celastrina idella	Satyrium saepium	Coenonympha tullia	Phyciodes tharos	Colias interior
Celastrina ladon	Satyrium sylvinus	Cyllopsis gemma	Poladryas arachne	Colias meadiii
Celastrina neglecta	Satyrium tetra	Cyllopsis pertepida	Poladryas minuta	Colias nastes
Celastrina neglectamajor	Satyrium titus	Danaus eresimus	Polygonia comma	Colias palaeno
Cupido amyntula	Strymon alea	Danaus gilippus	Polygonia faunus	Colias pelidne
Cupido comyntas	Strymon avalona	Danaus plexippus	Polygonia gracilis	Colias philodice
Echinargus isola	Strymon istapa	Dryas iulia	Polygonia interrogationis	Colias tyche
Electrostrymon angelia	Strymon martialis	Dymasia dymas	Polygonia oreas	Euchloe ausonides
Erora laeta	Strymon melinus	Emesis ares	Polygonia progne	Euchloe creusa
Erora quaderna	Zizula cyna	Emesis zela	Polygonia satyrus	Euchloe lotta
Eumaeus atala		Enodia anthedon	Satyrodes appalachia	Euchloe olympia
Euphilotes ancilla	**Nymphalidae**	Enodia portlandia	Satyrodes eurydice	Eurema boisduvaliana
Euphilotes battoides	Adelpha bredowii	Erebia callias	Siproeta stelenes	Eurema daira
Euphilotes baueri	Aglais milberti	Erebia disa	Speyeria adiaste	Eurema mexicana
Euphilotes bernadino	Agraulis vanillae	Erebia discoidalis	Speyeria aphrodite	Eurema salome
Euphilotes ellisi	Anartia jatrophae	Erebia fasciata	Speyeria atlantis	Ganyra josephina
Euphilotes enoptes	Aneae aidea	Erebia lafontainei	Speyeria callippe	Kricogonia lyside
Euphilotes glaucon	Aneae andria	Erebia mackinleyensis	Speyeria coronis	Nathalis iole
Euphilotes mojave	Anthanassa frisia	Erebia magdalena	Speyeria cybele	Neophasia terlooii
Euphilotes rita	Anthanassa texana	Erebia mancinus	Speyeria diana	Phoebis agarithe
Euphilotes spaldingi	Anthanassa tulcis	Erebia occulta	Speyeria edwardsii	Phoebis philea
Feniseca tarquinius	Apodemia duryi	Erebia pawloskii	Speyeria egleis	Phoebis sennae
Glaucopsyche lygdamus	Apodemia hepburni	Erebia rossii	Speyeria hesperis	Pieris marginalis
Habrodais grunus	Apodemia mejicanus	Erebia vidleri	Speyeria hydaspe	Pieris oleracea
Hemiargus ceraunus	Apodemia mormo	Erebia youngi	Speyeria idalia	Pieris rapae
Hypostrymon critola	Apodemia nais	Eunica monima	Speyeria mormonia	Pieris virginiensis
Leptotes cassius	Apodemia palmeri	Eunica tatila	Speyeria nokomis	Pontia beckerii
Leptotes marina	Apodemia virgulti	Euphydryas chalcedona	Speyeria zerene	Pontia occidentalis
Lycaena arota	Asterocampa celtis	Euphydryas editha	Texola elada	Pontia protodice
Lycaena cupreus	Asterocampa clyton	Euphydryas gillettii	Vanessa annabella	Pontia sisymbrii
Lycaena dione	Asterocampa leilia	Euphydryas phaeton	Vanessa atalanta	Pyrisitia dina
Lycaena dorcas	Biblis hyperia	Euptoieta claudia	Vanessa cardui	Pyrisitia lisa
Lycaena editha	Boloria alaskensis	Euptoieta hegesia	Vanessa virginiensis	Pyrisitia nise
Lycaena gorgon	Boloria alberta	Gyrocheilus patrobas		Pyrisitia proterpia
Lycaena helloides	Boloria astarte	Heliconius charithonia	**Papilionidae**	Zerene cesonia
Lycaena hermes	Boloria bellona	Hermeuptychia sosybius	Battus philenor	Zerene eurydice
Lycaena heteronea	Boloria chariclea	Junonia coenia	Battus polydamas	
Lycaena hyllus	Boloria epithore	Junonia evarete	Eurythides marcellus	
Lycaena mariposa	Boloria eunomia	Junonia genoveva	Papilio andraemon	
Lycaena nivalis	Boloria freija	Libytheana carinenta	Papilio androgeus	
Lycaena phlaeas	Boloria frigga	Limenitis archippus	Papilio aristodemus	
Lycaena rubidus	Boloria improba	Limenitis arthemis	Papilio brevicauda	
Lycaena xanthoides	Boloria kriemhild	Limenitis lorquini	Papilio canadensis	
Ministrymon azia	Boloria natazhati	Limenitis weidemeyerii	Papilio cresphontes	
Ministrymon clytie	Boloria polaris	Marpesia petreus	Papilio eurymedon	
Ministrymon leda	Boloria selene	Megisto cymela	Papilio glaucus	
Parrhasius m-album	Calephelis arizonensis	Megisto rubricata	Papilio indra	
Phaeostrymon alcestis	Calephelis borealis	Memphis glycerium	Papilio machaon	
Philotes sonorensis	Calephelis muticum	Mestra amymone	Papilio multicaudata	
Philotiella speciosa	Calephelis nemesis	Neominois ridingsii	Papilio ornythion	
Plebejus acmon	Calephelis perditalis	Neonympha areolata	Papilio palamedes	
Plebejus emigdionis	Calephelis virginiensis	Neonympha helicta	Papilio polyxenes	
Plebejus glandon	Calephelis wrighti	Neonympha mitchellii	Papilio rutulus	

Appendix S3: List of butterfly species of Europe.

**Lycaenidae**	Pseudaricia nicias	Erebia alberganus	Melanargia arge	Artogeia krueperi
Agriades glandon	Pseudophilotes abencerrag	Erebia calcaria	Melanargia galathea	Artogeia mannii
Agrodiaetus admetus	Pseudophilotes barbagiae	Erebia cassioides	Melanargia ines	Artogeia napi
Agrodiaetus ainsae	Pseudophilotes baton	Erebia christi	Melanargia lachesis	Artogeia rapae
Agrodiaetus amanda	Pseudophilotes bavius	Erebia claudina	Melanargia larissa	Colias alfacariensis
Agrodiaetus aroaniensis	Pseudophilotes panoptes	Erebia disa	Melanargia occitanica	Colias aurorina
Agrodiaetus damon	Pseudophilotes vicrama	Erebia embla	Melanargia russiae	Colias caucasia
Agrodiaetus dolus dolus	Quercusia quercus	Erebia epiphron	Melitaea aetherica	Colias chrysotheme
Agrodiaetus escheri	Satyrium acaciae	Erebia epistygne	Melitaea arduinna	Colias crocea
Agrodiaetus fabressei	Satyrium esculi	Erebia eriphyle	Melitaea cinxia	Colias erate
Agrodiaetus galloi	Satyrium ilicis	Erebia euryale	Melitaea diamina	Colias hecla
Agrodiaetus humedasae	Satyrium pruni	Erebia flavofasciata	Melitaea didyma	Colias hyale
Agrodiaetus iphigenia	Satyrium spini	Erebia gorge	Melitaea phoebe	Colias myrmidone
Agrodiaetus nephohiptamen	Satyrium w-album	Erebia gorgone	Melitaea trivia	Colias nastes
Agrodiaetus pyrenaicus	Scolitantides orion	Erebia hispania	Mellicta asteria	Colias palaeno
Agrodiaetus ripartii ripartii	Tarucus balcanicus	Erebia lefebvrei	Mellicta athalia	Colias phicomone
Agrodiaetus thersites	Tarucus theophrastus	Erebia ligea	Mellicta aurelia	Colotis evagore
Agrodiaetus violeta	Thecla betulae	Erebia manto	Mellicta britomartis	Elphinstonia penia
Albulina orbitulus	Tomares ballus	Erebia medusa	Mellicta deione	Euchloe ausonia
Aricia agestis	Tomares nogelii	Erebia melampus	Mellicta parthenoides	Euchloe belemia
Aricia artaxerxes	Turanana endymion	Erebia melas	Mellicta varia	Euchloe insularis
Aricia morronensis	Ultraaricia anteros	Erebia meolans	Minois dryas	Euchloe tagis
Cacyreus marshalli	Vacciniina optilete	Erebia mnestra	Neohipparchia fatua	Gonepteryx cleopatra
Callophrys avis	Zizeeria knysna	Erebia montana	Neohipparchia statilinus	Gonepteryx farinosa
Callophrys rubi		Erebia neoridas	Neptis rivularis	Gonepteryx rhamni
Celastrina argiolus	**Nymphalidae**	Erebia nivalis	Neptis sappho	Leptidea duponcheli
Chilades trochylus	Aglais urticae	Erebia oeme	Nymphalis antiopa	Leptidea morsei
Cupido carswelli	Apatura ilia	Erebia orientalis	Nymphalis polychloros	Leptidea sinapis-reali
Cupido lorquinii	Apatura iris	Erebia ottomana	Nymphalis vaualbum	Pieris brassicae
Cupido minimus	Apatura metis	Erebia palarica	Nymphalis xanthomelas	Pontia callidice
Cupido osiris	Aphantopus hyperantus	Erebia pandrose	Oeneis bore	Pontia chloridice
Cyaniris semiargus	Araschnia levana	Erebia pharte	Oeneis glacialis	Pontia daplidice-edusa
Eumedonia eumedon	Arethusana arethusa	Erebia pluto	Oeneis jutta	Zegris eupheme
Everes alcetas	Argynnis adippe	Erebia polaris	Oeneis norna	
Everes argiades	Argynnis aglaja	Erebia pronoe	Pararge aegeria	
Everes decoloratus	Argynnis elisa	Erebia rhodopensis	Polygonia c-album	
Glaucopsyche alexis	Argynnis laodice	Erebia scipio	Polygonia egea	
Glaucopsyche melanops	Argynnis niobe	Erebia sthennyo	Proclossiana eunomia	
Iolana iolas	Argynnis pandora	Erebia stirius	Proterebia afra	
Kretania eurypilus	Argynnis paphia	Erebia styx	Pseudochazara anthelea	
Kretania psylorita	Boloria aquilonaris	Erebia sudetica	Pseudochazara cingovskii	
Laeopsis roboris	Boloria graeca	Erebia triaria	Pseudochazara geyeri	
Lampides boeticus	Boloria napaea	Erebia tyndarus	Pseudochazara graeca	
Leptotes pirithous	Boloria pales	Erebia zapateri	Pseudochazara orestes	
Lycaena alciphron	Brenthis daphne	Eurodryas aurinia	Pseudotergumia fidia	
Lycaena candens	Brenthis hecate	Eurodryas desfontainii	Pyronia bathseba	
Lycaena dispar	Brenthis ino	Hipparchia alcyone	Pyronia cecilia	
Lycaena helle	Charaxes jasius	Hipparchia aristaeus	Pyronia tithonus	
Lycaena hippothoe	Chazara briseis	Hipparchia cretica	Satyrus actaea	
Lycaena ottomana	Chazara prieuri	Hipparchia fagi	Satyrus ferula	
Lycaena phlaeas	Clossiana chariclea	Hipparchia neomiris	Vanessa atalanta	
Lycaena thersamon	Clossiana dia	Hipparchia semele	Vanessa cardui	
Lycaena thetis	Clossiana euphorysne	Hipparchia syriaca	Vanessa virginiensis	
Lycaena tityrus	Clossiana freija	Hipparchia volgenis		
Lycaena virgaureae	Clossiana frigga	Hypodryas cynthia	**Papilionidae**	
Lysandra albicans	Clossiana polaris	Hypodryas iduna	Archon apollinus	
Lysandra bellargus	Clossiana selene	Hypodryas intermedia	Iphiclides podalirius	
Lysandra coridon	Clossiana thore	Hypodryas maturna	Papilio alexanor	
Lysandra hispana	Clossiana titania	Hyponephele lupina	Papilio hospiton	
Maculinea alcon	Coenonympha arcania	Hyponephele lycaon	Papilio machaon	
Maculinea arion	Coenonympha corinna	Inachis io	Parnassius apollo	
Maculinea nausithous	Coenonympha dorus	Issoria lathonia	Parnassius mnemosyne	
Maculinea telejus	Coenonympha gardetta	Kanetisa circe	Parnassius phoebus	
Meleageria daphnis	Coenonympha glycerion	Kirinia climene	Zerynthia cerisy	
Neolysandra coelestina	Coenonympha hero	Kirinia roxelana	Zerynthia polyxena	
Plebejus argus	Coenonympha leander	Lasiommata maera	Zerynthia rumina	
Plebejus argyrognomon	Coenonympha oedippus	Lasiommata megera		
Plebejus idas	Coenonympha pamphilus	Lasiommata petropolitan	**Pieridae**	
Plebejus pylaon	Coenonympha rhodopensis	Libythea celtis	Anthocharis belia	
Plebicula dorylas	Coenonympha thyrsis	Limenitis camilla	Anthocharis cardamines	
Plebicula golgus	Coenonympha tullia	Limenitis populi	Anthocharis damone	
Plebicula nivescens	Danaus chrysippus	Limenitis reducta	Anthocharis gruneri	
Polyommatus eroides	Danaus plexippus	Lopinga achine	Aporia crataegi	
Polyommatus eros	Erebia aethiopella	Maniola jurtina	Artogeia bryoniae	
Polyommatus icarus	Erebia aethiops	Maniola nurag	Artogeia ergane	

Appendix S4: Correlation of colour lightness data of North America and Europe.

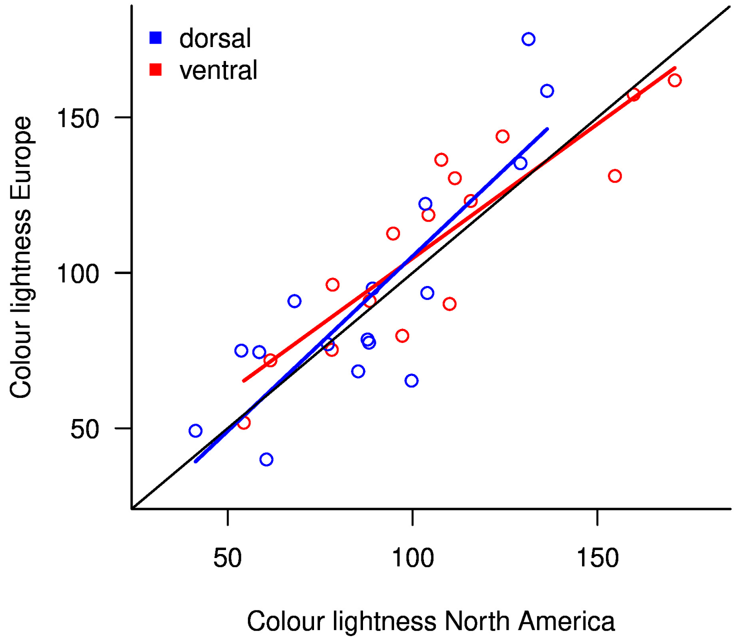

Dorsal and ventral images of 16 butterfly species were depicted in both Brock *et al.* (2003, ref. 47) and Tolman & Lewington (2009, ref. 48). Colour lightness values of the two sources were highly correlated (major axis regression: dorsal $r^2 = 0.96$, ventral $r^2 = 0.98$, $p < 0.001$) and the slopes did not differ from 1 (lines forced through the origin, $p > 0.05$, R package *smatr*).

Species: *Coenonympha tullia, Colias hecla, Colias nastes, Colias palaeno, Danaus plexippus, Erebia disa, Lycaena phlaeas, Nymphalis antiopa, Nymphalis vaualbum, Oeneis bore, Oeneis jutta, Papilio machaon, Plebejus idas, Vanessa atalanta, Vanessa cardui, Vanessa virginiensis.*

Appendix S5: Number of species and grid cells (butterfly assemblages) in North America and Europe. The number of grid cells with at least five species, which were used in the statistical analysis, are also given. Number of species does not differ between datasets containing grids cells with all species and at least five species, except for European Papilionids where one species (*Papilio hospiton*) dropped out.

Family	North America			Europe		
	Species	Grid cells		Species	Grid cells	
		All species	≥ 5 species		All species	≥ 5 species
All	330	9,220	8,499	326	1,939	1,840
Lycaenidae	96	8,686	6,526	98	1,870	1,546
Nymphalidae	164	9,160	8,274	177	1,899	1,757
Papilionidae	21	6,824	2,364	11	1,418	169
Pieridae	49	9,128	5,971	40	1,856	1,465

Appendix S6: Maps of environmental variables.

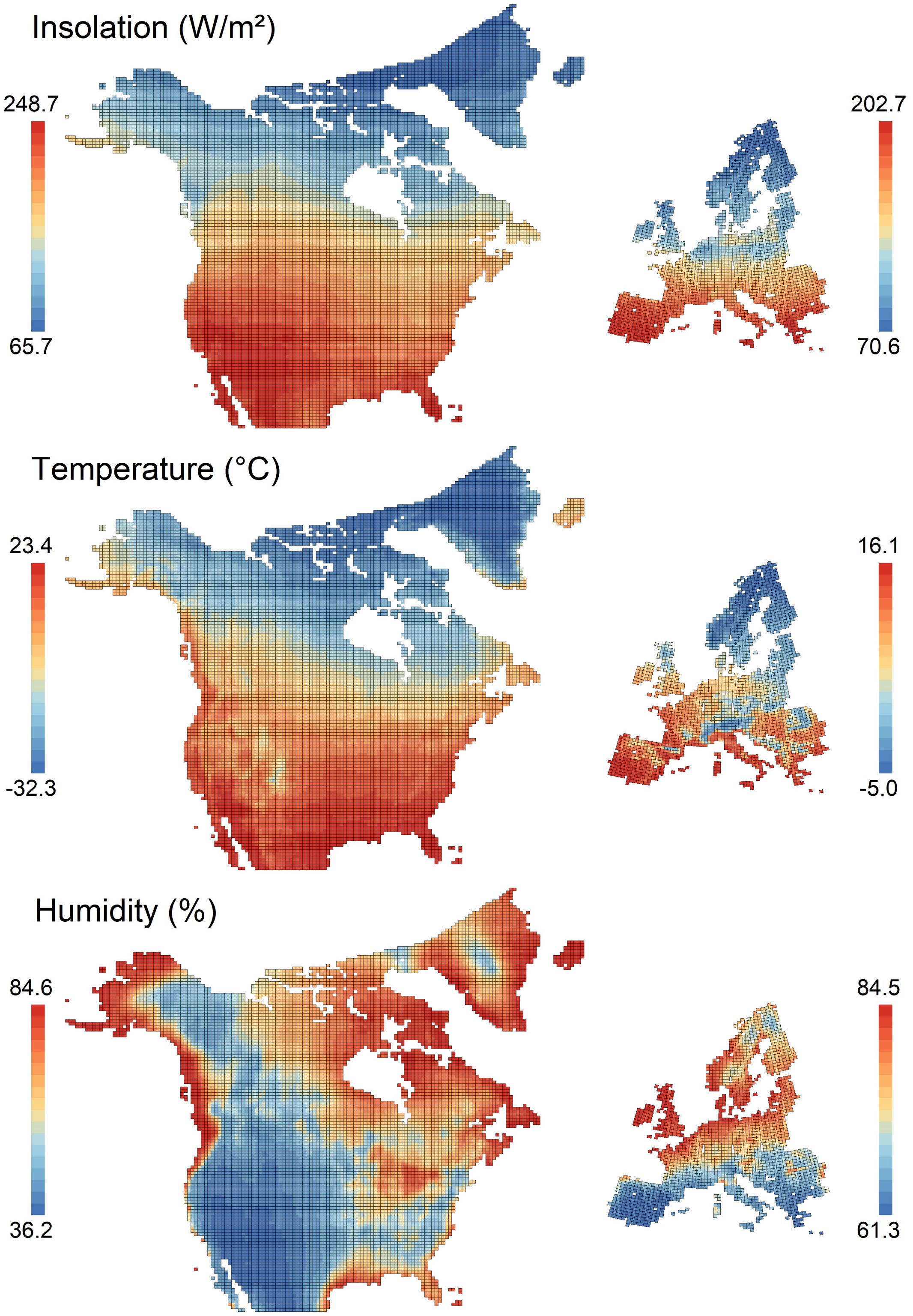

Appendix S7: The effect of the environmental variables insolation, temperature and humidity on the colour lightness of butterfly families. Shown are scatterplots and regressions.

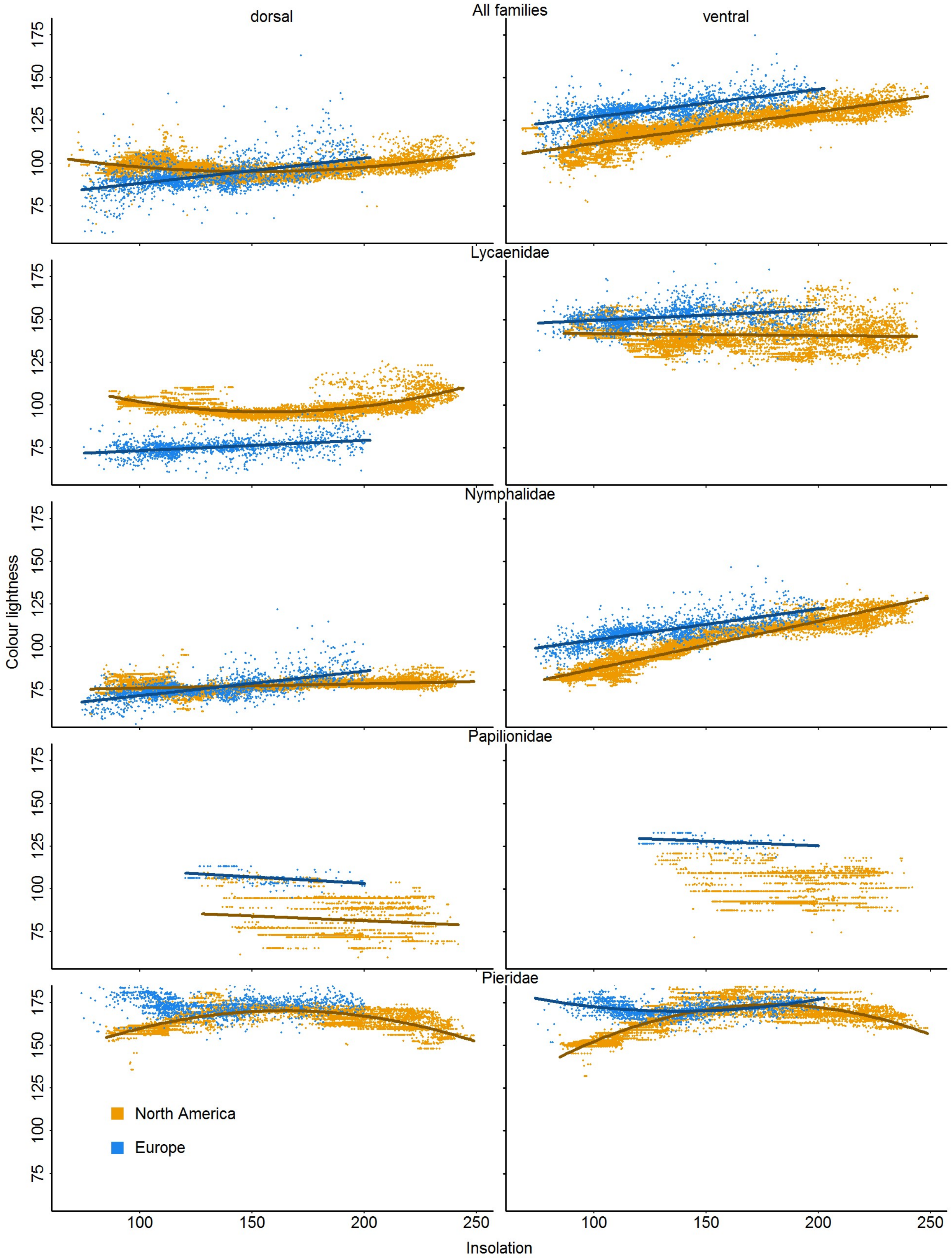

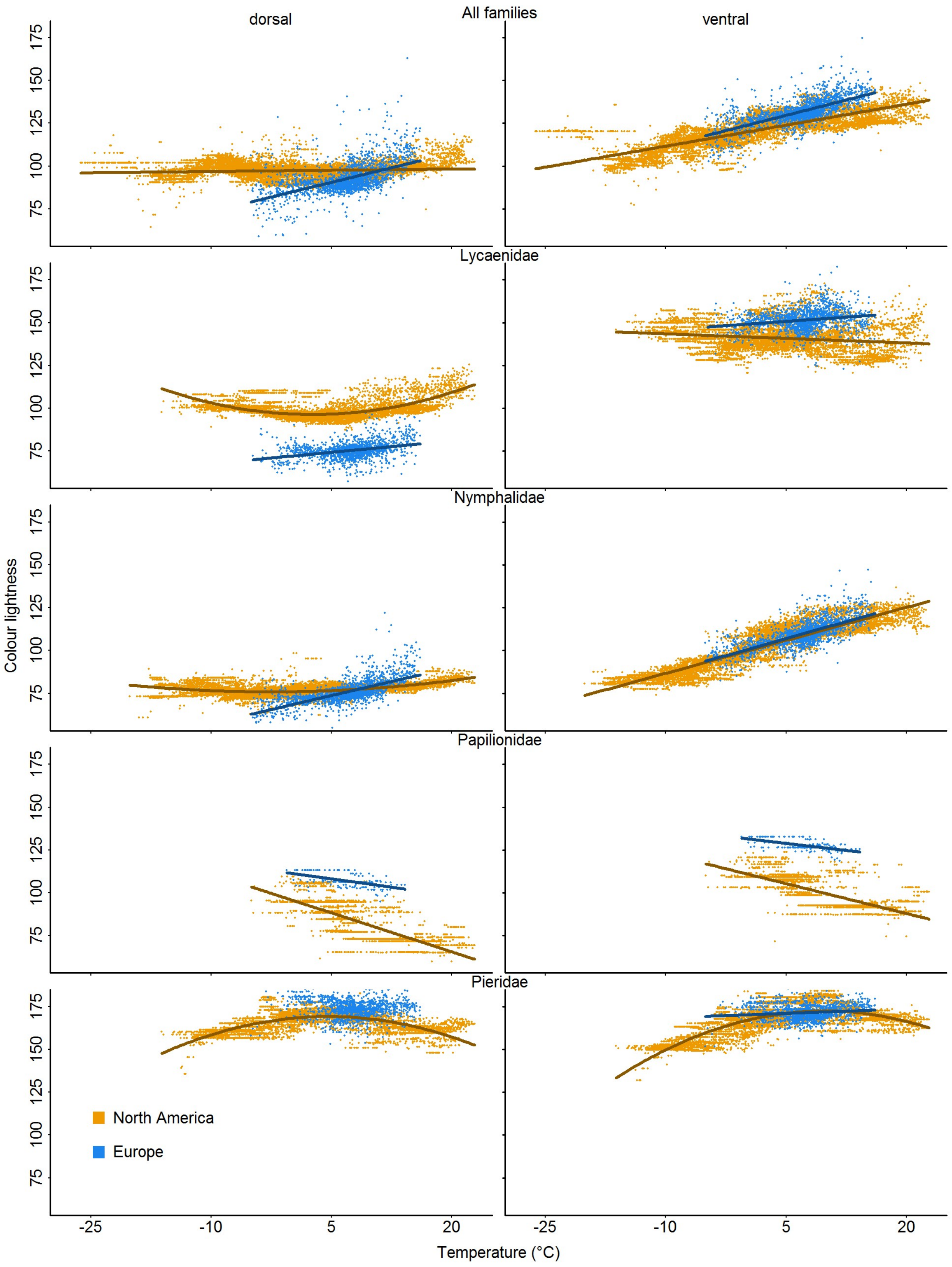
All families
dorsal
ventral
Lycaenidae
Nymphalidae
Papilionidae
Pieridae
Colour lightness
75
100
125
150
175
North America
Europe
-25
-10
5
20
Temperature (°C)

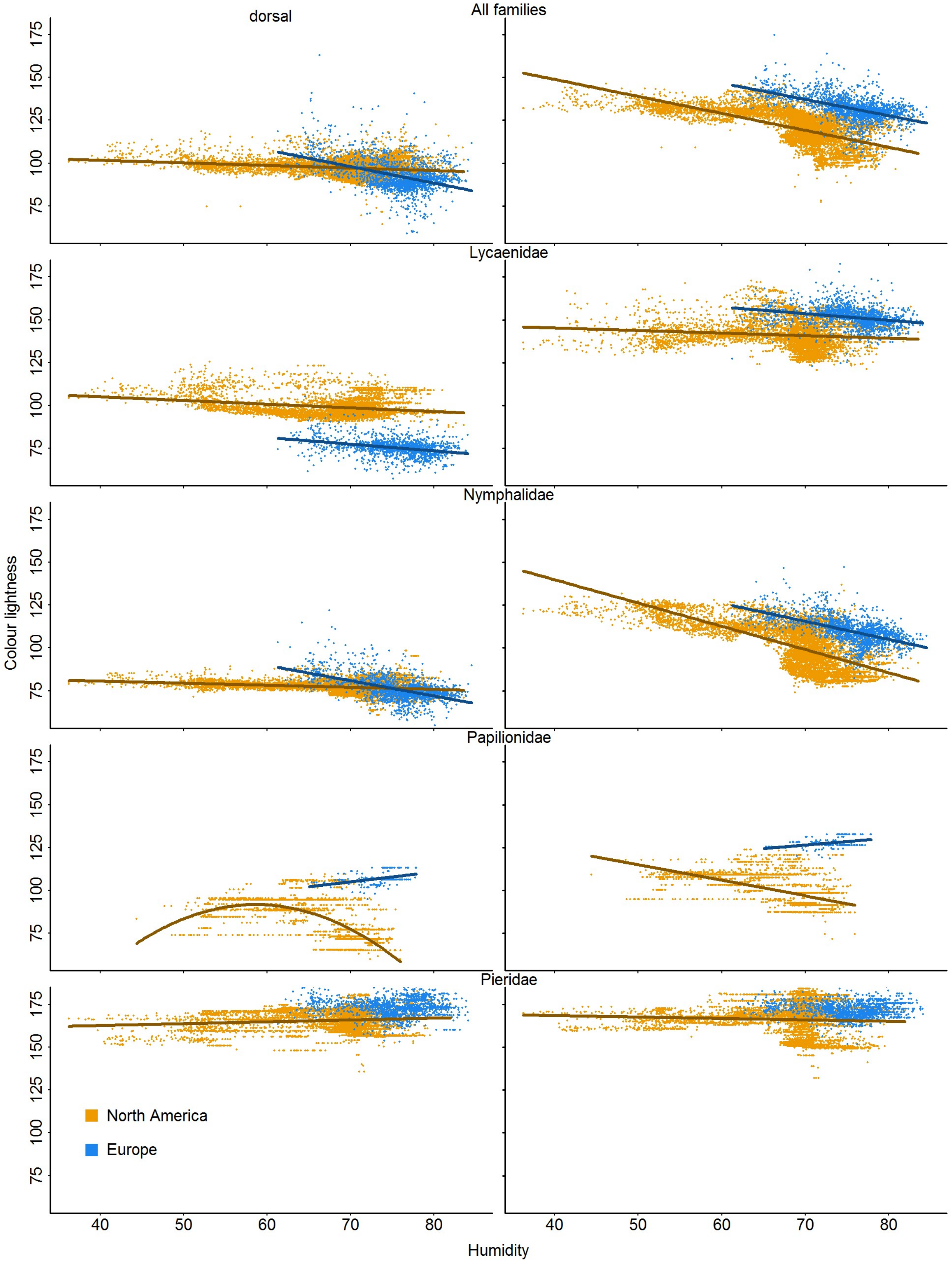
dorsal
All families
Lycaenidae
Nymphalidae
Papilionidae
Pieridae
Colour lightness
Humidity
North America
Europe
175
150
125
100
75
40
50
60
70
80

Appendix S8: Statistics of linear and quadratic generalized least-squares regressions between the colour lightness of butterfly assemblages across North America and Europe and the three potential environmental drivers temperature, insolation and humidity. Models were fitted with a Gaussian spatial correlation structure of geographical coordinates to account for spatial autocorrelation. Models were computed for the complete datasets of each continent and separately for the major butterfly families. Direction of the effects (+/−) and r^2 values are given for each model. If Δr^2 (quadratic r^2 − linear r^2) was > 0.1, a quadratic shape (U- or hump-shaped) of the relationship was assumed; otherwise a linear shape was assumed, as also indicated by the inserted pictograms. Only assemblages with at least five species were included in this analysis. Level of significance was set to 0.001 (n.s. = not significant). Highest r^2 values for the assumed relationship in each family and continent are highlighted in bold. Different shapes of the relationships compared with the results presented in Table 1 of the main text are highlighted in grey.

Variable	Family	Side	North America Linear r^2	North America Quadratic r^2	North America Δ r^2	North America Shape	Europe Linear r^2	Europe Quadratic r^2	Europe Δ r^2	Europe Shape
INSOLATION	All	Dorsal	+ 0.00	+ 0.07	0.07	/	+ 0.15	+ 0.17	0.01	/
		Ventral	**+ 0.42**	− 0.43	0.01	/	**+ 0.29**	− 0.29	0.00	/
	Lycaenidae	Dorsal	+ 0.02	+ 0.15	0.13	∪	**+ 0.08**	+ 0.09	0.01	/
		Ventral	− 0.00	+ 0.03	0.03	\	**+ 0.05**	− 0.07	0.02	/
	Nymphalidae	Dorsal	**+ 0.05**	+ 0.09	0.04	/	+ 0.27	+ 0.27	0.00	/
		Ventral	**+ 0.68**	− 0.71	0.03	/	**+ 0.36**	− 0.37	0.01	/
	Papilionidae	Dorsal	− 0.01	+ 0.03	0.02	\	− 0.11	+ 0.11	0.00	\
		Ventral	− 0.00	+ 0.05	0.05	\	− 0.09	− 0.09	0.00	\
	Pieridae	Dorsal	+ 0.00	**− 0.25**	0.25	∩	n.s.	+ 0.04	0.04	
		Ventral	+ 0.17	**− 0.44**	0.27	∩	n.s.	+ 0.06	0.05	
TEMPERATURE	All	Dorsal	**+ 0.01**	+ 0.03	0.02	/	**+ 0.17**	+ 0.22	0.04	/
		Ventral	+ 0.34	− 0.34	0.00	/	+ 0.29	+ 0.29	0.01	/
	Lycaenidae	Dorsal	+ 0.02	**+ 0.16**	0.14	∪	+ 0.07	+ 0.11	0.04	/
		Ventral	**− 0.01**	+ 0.01	0.00	\	+ 0.03	− 0.03	0.00	/
	Nymphalidae	Dorsal	+ 0.04	+ 0.10	0.06	/	**+ 0.29**	+ 0.33	0.04	/
		Ventral	+ 0.57	− 0.57	0.00	/	+ 0.33	+ 0.34	0.01	/
	Papilionidae	Dorsal	**− 0.24**	+ 0.24	0.00	\	**− 0.15**	− 0.16	0.01	\
		Ventral	**− 0.19**	+ 0.19	0.00	\	**− 0.15**	n.s.	0.00	\
	Pieridae	Dorsal	+ 0.00	− 0.21	0.21	∩	n.s.	+ 0.01	0.01	
		Ventral	+ 0.19	− 0.35	0.16	∩	**+ 0.01**	+ 0.02	0.01	/
HUMIDITY	All	Dorsal	− 0.01	+ 0.02	0.01	\	− 0.10	+ 0.14	0.04	\
		Ventral	− 0.19	− 0.20	0.01	\	− 0.14	+ 0.15	0.01	\
	Lycaenidae	Dorsal	− 0.02	+ 0.05	0.03	\	− 0.06	+ 0.07	0.00	\
		Ventral	− 0.01	+ 0.01	0.00	\	− 0.04	− 0.05	0.01	\
	Nymphalidae	Dorsal	− 0.02	+ 0.02	0.00	\	− 0.15	+ 0.18	0.02	\
		Ventral	− 0.24	− 0.25	0.01	\	− 0.17	+ 0.17	0.00	\
	Papilionidae	Dorsal	− 0.10	− 0.14	0.04	\	+ 0.10	+ 0.11	0.01	/
		Ventral	− 0.11	− 0.13	0.02	\	+ 0.07	+ 0.09	0.01	/
	Pieridae	Dorsal	+ 0.00	− 0.01	0.01	/	n.s.	n.s.	0.00	
		Ventral	− 0.00	− 0.01	0.01	\	n.s.	+ 0.02	0.02	

Appendix S9: Comparisons between dorsal and ventral colour lightness.

Family	Study region	Dorsal	Ventral	df	t	p
All	North America	98.12	127.55	657.89	-10.61	< 0.001
	Europe	89.22	130.95	647.38	-14.40	< 0.001
Lycaenidae	North America	99.21	140.57	156.82	-11.03	< 0.001
	Europe	78.92	159.11	184.64	-25.30	< 0.001
Nymphalidae	North America	79.44	109.84	265.19	-10.66	< 0.001
	Europe	76.85	108.08	351.72	-10.83	< 0.001
Papilionidae	North America	80.86	104.86	38.08	-2.37	< 0.05
	Europe	100.82	123.68	19.85	-3.63	< 0.01
Pieridae	North America	165.35	163.03	95.42	0.51	0.608
	Europe	166.05	165.20	71.79	0.16	0.88

Appendix S10: Histograms of colour lightness values.

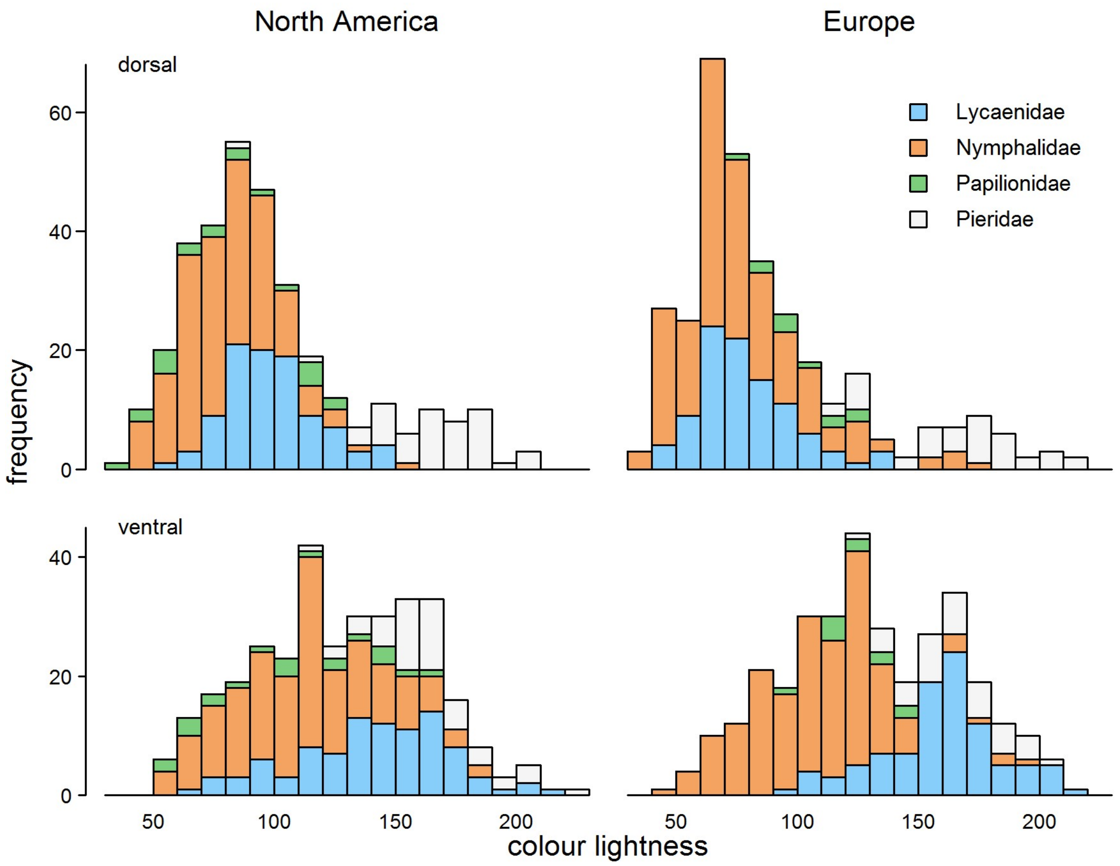

Distribution of colour lightness values separated according to study region, dorsal and ventral side, and butterfly families.
